# Functionalization of Bacterial Cellulose with the Antimicrobial Peptide KR-12 via Chimerical Cellulose-Binding Peptides

**DOI:** 10.3390/ijms25031462

**Published:** 2024-01-25

**Authors:** Elizabeth M. van Zyl, Jeannine M. Coburn

**Affiliations:** Department of Biomedical Engineering, Worcester Polytechnic Institute, Worcester, MA 01609, USA

**Keywords:** KR-12, antibacterial activity, bacterial cellulose, antimicrobial peptides, cellulose binding peptides, wound dressing

## Abstract

Bacterial-derived cellulose (BC) has been studied as a promising material for biomedical applications, including wound care, due to its biocompatibility, water-holding capacity, liquid/gas permeability, and handleability properties. Although BC has been studied as a dressing material for cutaneous wounds, to date, BC inherently lacks antibacterial properties. The current research utilizes bifunctional chimeric peptides containing carbohydrate binding peptides (CBP; either a short version or a long version) and an antimicrobial peptide (AMP), KR-12. The secondary structure of the chimeric peptides was evaluated and confirmed that the α-helix structure of KR-12 was retained for both chimeric peptides evaluated (Long-CBP-KR12 and Short-CBP-KR12). Chimeric peptides and their individual components were assessed for cytotoxicity, where only higher concentrations of Short-CBP and longer timepoints of Short-CBP-KR12 exposure exhibited negative effects on metabolic activity, which was attributed to solubility issues. All KR-12-containing peptides exhibited antibacterial activity in solution against *Escherichia coli* (*E. coli*) and *Pseudomonas aeruginosa* (*P. aeruginosa*). The lipopolysaccharide (LPS) binding capability of the peptides was evaluated and the Short-CBP-KR12 peptide exhibited enhanced LPS-binding capabilities compared to KR-12 alone. Both chimeric peptides were able to bind to BC and were observed to be retained on the surface over a 7-day period. All functionalized materials exhibited no adverse effects on the metabolic activity of both normal human dermal fibroblasts (NHDFs) and human epidermal keratinocyte (HaCaT) epithelial cells. Additionally, the BC tethered chimeric peptides exhibited antibacterial activity against *E. coli*. Overall, this research outlines the design and evaluation of chimeric CBP-KR12 peptides for developing antimicrobial BC membranes with potential applications in wound care.

## 1. Introduction

Increasing concern lies in the danger of chronic wounds due to an aging population and the widespread rise of antibiotic resistance [1]. In the US, chronic wounds affect 2.4–4.5 million people, resulting in a yearly direct Medicare cost of over USD 13.5 billion [1,2] Chronic wounds, wounds that fail to progress through normal healing processes within 3 months, generally develop due to systemic factors such as age and disease state (diabetes, keloids, hereditary healing disorders), medications, and overall nutrition [3,4]. These wounds are marked by senescent fibroblast and keratinocyte cell populations due to increased oxidative stress, elevated H_2_O_2_-producing enzymes, a lack of antioxidant pathways, and subsequent high levels of reactive oxygen species (ROS) shortly after injury [5,6,7,8,9,10]. A further lack of wound closure and wound healing activity allows invading pathogens to access the wound site and flourish in the moist nutrient-rich wound bed [1]. Wound infection control is currently addressed with wound debridement and the application of antiseptics or antibiotics, although these are toxic to mammalian cells and promote the development of antibiotic-resistant bacteria [11]. Antiseptics, such as chlorhexidine and iodine, inhibit fibroblast proliferation and migration, impeding normal wound-healing progression [12]. Additionally, chlorohexidine resistance in specific bacterial species and strains has been found to be synonymous with increased antibiotic resistance [13,14]. Alternative methods for addressing wound infections include the use of silver-based gels or materials. Silver has been shown to upregulate pro-MMP-9 in stimulated cells, elevated levels of which are already known to be present in chronic wounds. This is believed to be responsible for preventing wound healing through growth factor degradation and the degradation of provisional extracellular matrix (ECM) laid down during early wound healing steps [12,15]. Overall, these antimicrobial approaches are associated with hindered cellular proliferation, leading to a need for an antimicrobial agent that overcomes antibiotic resistance and promotes healing [16].

Cationic AMPs, such as human cathelicidin LL-37, offer a promising alternative, given the growing concerns about antibiotic resistance [17]. Antimicrobial peptides (AMPs) are small molecular weight proteins that have broad spectrum antimicrobial activity against invading bacteria, viruses, and fungi [18,19]. AMPs are part of the host’s natural immune system and are generally positively charged and have both hydrophobic and hydrophilic sides to enable aqueous solubility and lipid membrane penetration capability [18,19]. The antimicrobial activity of AMPs is achieved mainly through non-receptor mediated damage to the bacterial membrane. This non-specific physical disruption of the microbial cell wall is driven by the electrostatic interaction between the positively charged AMP and negatively charged bacterial membrane [20]. This interaction increases membrane permeability and eventually leads to cell lysis [21]. Though effective as an antibacterial agent, LL-37 exhibits cytotoxicity at concentrations higher than 10 μM and has limited in vivo stability [16,17,22,23,24]. Additionally, its long peptide chain makes it susceptible to proteolytic degradation and in some cases *Staphylococcus aureus* (*S. aureus*) has been observed to develop small colony variants that develop resistance mechanisms to LL-37, such as transmembrane potential reduction, unsaturation of lipids, and metabolic alteration [16,17,20,22,25,26]. The shortest antimicrobial motif of LL-37, KR-12, overcomes these limitations [26]. KR-12 exhibits various bioactive properties such as broad antibacterial activity against both Gram-negative and Gram-positive bacteria, neutralization of lipopolysaccharides (LPS), modulation of inflammatory response, and possible promotion of wound closure through epithelialization functions such as epithelial cell migration, proliferation, and differentiation [26,27,28,29,30,31]. Though the narrow therapeutic ratio and susceptibility to proteolysis of AMPs in solution hinders their clinical use, AMP immobilization has been shown to decrease mammalian cell cytotoxicity and increase in vitro stability without compromising the AMP’s antimicrobial activity [16,22,32].

BC has been studied for wound dressing applications due to its biocompatibility, water-holding capacity, liquid/gas permeability, and handleability properties [4,33,34]. With BC’s extensive exploration for use as a wound dressing and the more recent incorporation of wound visualization capabilities, the lack of antibacterial surface properties remains to be addressed [35,36]. Cellulose binding peptides (CBPs), also called cellulose-binding domains/modules, interact with and bind to the cellulose surface to bring the hydrolytic region of cellulase enzymes into proximity with the cellulose surface [37]. CBPs provide a mechanism for immobilizing AMPs to the surface of BC, overcoming the limitation attributed to mammalian cytotoxicity and aiding in the prevention of wound infection [23,38,39,40,41]. Recent advancements in this field include Weishaupt et al.’s use of a short CBP sequence to non-covalently functionalize cellulose with the antimicrobial ligand tet009, and Barbosa et al.’s use of a full cellulose-binding domain (CBD) to tether the antimicrobial hexapeptide MP196 to cellulose-based materials [37,42].

To further develop bifunctional chimeric peptide sequences for the non-covalent functionalization of BC, this work evaluates two different CBP sequences. The first CBP evaluated (Long-CBP) was engineered by Khazanov et al. by modifying the sequence and structure of cellulose-binding domains from *Trichoderma reesei* cellobiohydrolase I [38]. Specifically, the Long-CBP is comprised of residues 4–8, 25–36 from the complete CBD, with the following engineered mutations: H4G, Y5W, Y31W, C35T, insT35a, and L36P. Khazanov et al. determined these engineered mutations resulted in a CBP with a higher cellulose-binding affinity (1.45 ± 0.30 × 10^6^ M^−1^) and increased maximal surface coverage (6.77 ± 0.10 µmol/g) compared to the complete CBD (0.94 ± 0.15 × 10^6^ M^−1^ and 5.07 ± 0.05 µmol/g) [38]. The second CBP evaluated in this work (Short-CBP) was initially identified and characterized by Guo et al. using phage display [43]. The Short-CBP was found to have a binding affinity to crystalline cellulose of ~10^5^ M^−1^, span approximately five glucose rings in length, and exhibit cellulose binding through CH/π interactions and hydrogen bonding between peptide sidechains and cellulose [43]. Within the realm of CBDs, this heptapeptide sequence has been described as a minimal consensus amino acid sequence [37,43].

Though this is the first reported use of CBPs to tether KR-12 to a material surface, alternative covalent and electrostatic-based methods have been explored with varying success. Diosa et al. studied the antimicrobial activity and proteolytic stability of KR-12 absorbed to a chitosan–silica hybrid material. Adsorbed peptide exhibited decreased antibacterial activity due to steric hindrance, but increased protection from proteolytic activity due to selective interactions with the solid surface [44]. Liu et al. conjugated KR-12 onto eggshell membranes and reported less than 3% bacterial survival of *E. coli*, *S. aureus*, and methicillin-resistant *Staphylococcus aureus* (MRSA). Further, HaCaT cells, a keratinocyte cell line, cultured on KR-12 functionalized eggshell membranes exhibited increased proliferation levels compared to unfunctionalized eggshell membranes [45]. Blasi-Romero et al. recently investigated covalent methods to immobilize KR-12, specifically comparing EDC/NHS coupling chemistry, thiol–ene click chemistry, and Cu(I)-catalyzed azide-alkyne cycloaddition [30]. Though effective at immobilizing KR-12, covalent methods exhibit limitations, such as requiring initial carboxymethylation of cellulose material for EDC/NHS coupling and thiol–ene click chemistry, and increased mammalian cytotoxicity for Cu(I)-catalyzed azide-alkyne cycloaddition. Additionally, added processing and wash steps are required to prevent cytotoxicity caused by the catalysts and unreacted reactants. Though KR-12 provided an excellent means of providing antibacterial activity without the concern of antibiotic resistance, more effective immobilization methods are required to maintain peptide function.

In this work, we non-covalently tethered an antimicrobial peptide (KR-12) to the surface of BC by harnessing the cellulose-binding affinity of CBP sequences and overcoming the processing steps and cytotoxic complication associated with covalent conjugations [30,46]. The peptide structure, mammalian cytotoxicity, antibacterial capabilities, endotoxin binding, and cellulose-binding properties of the soluble chimeric peptides (denoted as Long-CBP-KR12 and Short-CBP-KR12) were assessed. Immobilized peptide bioactivity was further assessed through functionalized BC cytotoxicity and antibacterial properties.

## 2. Results and Discussion

### 2.1. Peptide Structure and Helix Analysis

Peptide sequences were designed with three main components: a CBP domain, a flexible linker, and the AMP KR-12 (Table 1). Two different CBP sequences were chosen, denoted as Long-CBP and Short-CBP. From the literature, the Long-CBP was engineered by modifying the sequence and structure of cellulose-binding domains from *Trichoderma reesei* cellobiohydrolase I, while the Short-CBP was originally identified as a minimal consensus amino acid sequence via phage display [38,43]. A glycine- and serine-rich linker was used to allow rotational freedom for the two active domains, and KR-12 was immobilized via the N-terminus to match the immobilization direction previously presented for LL-37 and LL-37 fragments similar to KR-12 [16,23,47,48,49]. Ultimately, the two chimeric peptides were designed (Long-CBP-KR12 and Short-CBP-KR12) and evaluated in comparison with the two CBP-only peptides and KR-12.

**Figure 1:**
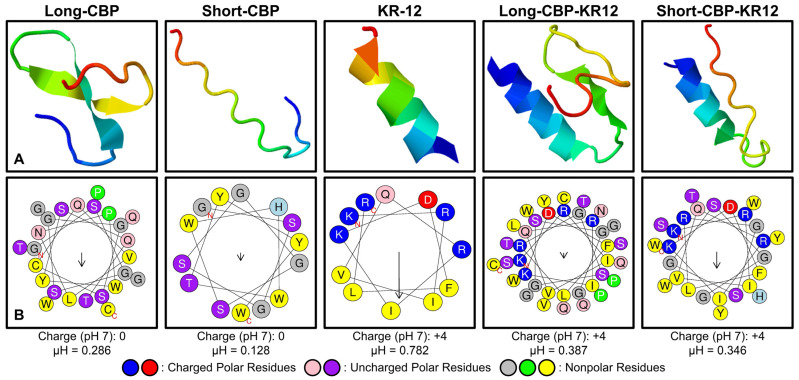
Predicted peptide structure and helix polarity delineation. (**A**) Peptide structural prediction in silico using the online I-TASSER server. (**B**) Helical wheel diagrams generated using the HeliQuest platform. Arrows represent the helical hydrophobic moment of each peptide.

Three dimensional models of each chimeric peptide sequence and their individual peptide components were generated with the understanding that the main bioactivity observed from cationic AMPs are due to the peptide’s α-helix structure and amphiphilicity. The Iterative Threading ASSEmbly Refinement (I-TASSER) server was used as an in silico method for predicting the secondary structure of the peptide sequences (Table 1, Figure 1A). The highest ranked model for each peptide submitted for analysis is shown. In each model, the red end correlates to the C-terminal, and the blue end correlates to the N-terminal of the sequence. Due to the server’s minimum amino acid sequence requirements, the Short-CBP sequence was submitted including the flexible linker sequence; the same was performed for the Long-CBP for consistency. For the Short-CBP, a secondary structure of beta strands and random coils was predicted; however, the Long-CBP, without the AMP linked, has a small region predicted to be an α-helix structure. The predicted α-helix structure is likely due to the proline residues found in the sequence that are known to be an effective inducer of helical hairpin turns [50,51]. In agreement with other studies that have used the I-TASSER server to predict the secondary structure, KR-12 was predicted to have an α-helix structural conformation [52]. This helical prediction was conserved in both the Long-CBP-KR12 and Short-CBP-KR12 sequences. Interestingly, the region of the Long-CBP is predicted to have an inherent α-helix structure, increases in length when linked to KR-12. Proline residues, though effective helical hairpin inducers, depend on a minimal amino acid length requirement. Previous studies have found that proline is most effective at inducing the α-helix conformation when found in the middle of a 40 amino acid-long sequence [51]. The Long-CBP-KR12 is a 37 amino acid-long peptide where proline is found at position 25. The I-TASSER server reports and produces up to five models of the predicted peptide structure, based on the generation of large ensembles of structural conformations and subsequent pair-wise analysis for structural similarities.

To further investigate the relationship between the α-helicity, net charge, hydrophobic moment, amino acid location, and amphiphilicity of the peptides, HeliQuest was used to generate helical wheel diagrams (Figure 1B). KR-12’s antibacterial activity is primarily driven by the amino acid location and resulting amphiphilicity of the α-helix structure [26]. When evaluating sequences with little to no helicity, e.g., the Long-CBP and Short-CBP sequences through the HeliQuest platform, there was no clear observational divide between the locations of polar and non-polar residues. KR-12, a sequence known to be amphiphilic, produced a helical wheel diagram that clearly portrayed the delineation between polar and non-polar residues. The helical wheel diagram for Long-CBP-KR12 and Short-CBP-KR12 indicated that this delineation is maintained in the chimeric peptide sequences. The hydrophobic moment (µH), a quantifiable measure of amphiphilicity using the vector sum of all hydrophobic indices divided by the number of residues, is only meaningful for peptides with an α-helix structure and has been shown to be more important for antibacterial activity than the degree of helicity [53,54]. KR-12 has a hydrophobic moment of 0.782; this is significantly higher than LL-37’s hydrophobic moment of 0.440 [28]. The Long-CBP-KR12 and Short-CBP-KR12 have hydrophobic moments of 0.387 and 0.346, respectively, indicating that the CBP and linker have significant effects on the amphiphilicity of the peptide sequences. Though this indicates the potential for decreased antibacterial activity, from the design of these chimeric peptides and the structural models (Figure 1A), it is known that a portion of the sequence is not an α-helix and is not designed to interact with bacterial membranes. This reduces the concern over decreased antibacterial activity solely due to a reduction in hydrophobic moment. The positive charge of cationic AMPs is essential for the initial electrostatic interactions between the peptide and the bacterial membrane [55]. The charge of the CBPs on their own were seen to be neutral at pH 7, while both chimeric sequences adopted the +4 charge exhibited by KR-12.

**Figure 2:**
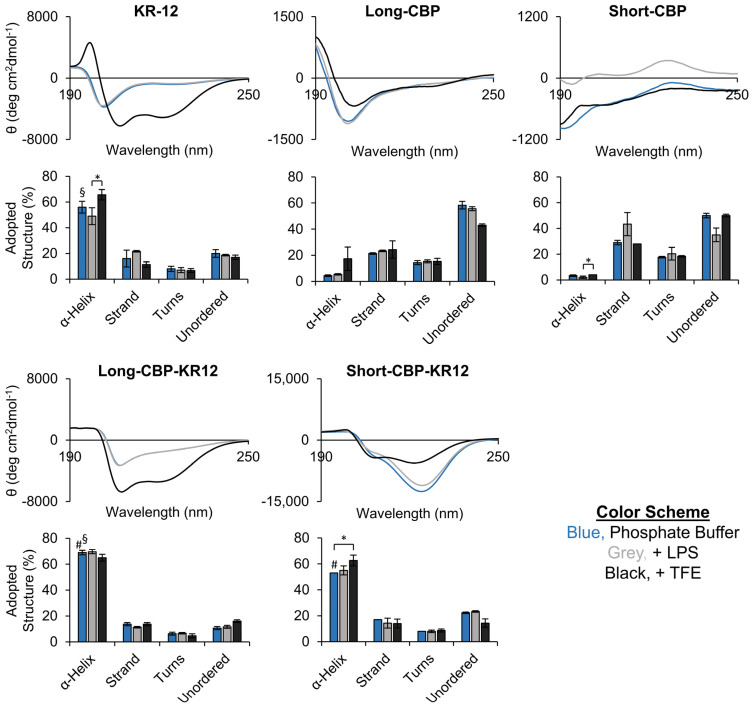
Peptide secondary structure evaluation via CD spectroscopy. CD spectra of each peptide in phosphate buffer (blue), buffer with 0.2% LPS (grey), and buffer with 50% TFE (black). Data are presented as the mean spectra from three independent readings (N = 3). Adopted structures were obtained from each independent reading. Statistical differences between α-Helix structure comparing across solution conditions was determined by an ANOVA followed by the Tukey’s HSD test (* *p* < 0.05). Statistical differences between α-Helix structure of KR-12 and chimeric KR-12 peptides were determined by an ANOVA followed by the Tukey’s HSD test (§ *p* < 0.01 between KR-12 and Short-CBP-KR12; # *p* < 0.01 between Short-CBP-KR12 and Long-CBP-KR12).

To further assess the secondary structure of each peptide experimentally, circular dichroism (CD) spectroscopy was performed using 10 mM phosphate buffer (standard aqueous environment), buffer containing 0.1% LPS (representative aqueous environment at the bacterial membrane interface), and buffer containing 50% trifluoroethanol (TFE; induces the α-helix conformation) (Figure 1B). The characteristics of an α-helix secondary structure in CD spectra are two minima values at 208 nm and 222 nm, and a positive band around 192 nm [26]. As increasing amounts of random coil/unordered structure is adopted, the minimum at 222 nm becomes shallower and the minimum at 208 nm shifts towards lower wavelengths. Most AMPs assume a highly structured secondary structure upon interacting with the bacterial membrane [56]. KR-12’s parent AMP, LL-37, however, can adopt an α-helix structure in the presence of chlorine, phosphate, sulfate, and bicarbonate ions at millimolar concentrations [57]. This process is described as the ability of the ions to “salt out” the non-polar residues of the peptide and induce secondary structural conformation [57]. Though not as widely reported, it is not surprising nor a new observation that the CD spectra of KR-12 in phosphate buffer and phosphate buffer containing LPS look very similar (Figure 2). In both solutions, KR-12 was seen to produce spectra with a small minimum around the 222 nm range and a larger minimum closer to 206 nm. In comparison, the spectra for KR-12 in phosphate buffer containing TFE had more pronounced minima at both the 222 nm and 206 nm wavelengths. CD spectra results were run through DichroWeb to determine the adopted secondary structure of each peptide sequence (Figure 2). For KR-12, a significant difference in the adopted percentage of the α-helix structure was observed between KR-12 in phosphate buffer containing LPS or TFE with 49% ± 7% and 56% ± 6% helicity, respectively. It should be noted that the percentage of α-helix structure observed in phosphate buffer containing TFE does not represent the true secondary structure of the peptide, but rather the relative tendency of the peptide to form an α-helix structure [53].

The Long-CBP in phosphate buffer containing or not containing LPS adopted a more random coil, or unordered, conformation with a minimum in the 195 nm region. The Long-CBP in phosphate buffer containing TFE exhibited an α-helix structure with a minimum returning around the 222 nm range. This structural change was confirmed as the adopted structure, indicating a ~10% drop in unordered structure and a corresponding ~10–15% increase in adopted α-helix structure. The Short-CBP comprised predominantly random coil/unordered and strand conformations in all solutions tested, consistent with the computationally predicted structure (Table 1). Similar findings were reported by Guo et al., where the peptide’s short amino acid sequence prevented higher order structures from forming [43].

Both Long-CBP-KR12 and Short-CBP-KR12 indicated high helicity in all solutions. When comparing the percent helicity using phosphate buffer, the Long-CBP-KR12 adopted a higher α-helix conformation of 69% ± 2% compared to both KR-12 and Short-CBP-KR12 with 56% ± 5% and 53% ± 0%, respectively. Interestingly, the spectra for Long-CBP-KR12 in phosphate buffer with or without LPS had shallow minimum values at 222 nm, characteristic of a more random coil/unordered structure; however, the minimum was not shifted to lower wavelengths as seen in the KR-12 spectra. For this reason, the adopted helicity percentage between Long-CBP-KR12 in all three buffered solutions was unchanged despite seemingly different-appearing spectra. Similarly for Short-CBP-KR12, a larger minimum in the 222 nm region for the peptide in buffer and buffer with LPS was observed, but not for the peptide in buffer with TFE, and no shift in location of the minimum at 208 nm. Overall, the experimental determination of the peptide conformation and secondary structure matched the qualitative modeling, and the potential of an α-helix conformation was retained in both chimeric peptide sequences.

With the understanding that bioactivity of AMPs, such as KR-12, is complex and relates to many factors including helicity and hydrophobic moment, Table 2 contains a summary of the physiochemical properties for each peptide sequence studied in this work. For consistency with the modeling data, all CBP sequences were assessed with the flexible linker attached. Generally, AMPs have molecular weights less than 10 kDa, isoelectric points (pI) greater than or equal to 10, positive net charges, and an instability index under 40 [58]. All peptides containing KR-12 fell within the ideal molecular weight and instability index range and had positive net charges. The isoelectric point, or pH where the peptide carries no net charge, was above 10 for KR-12 and the Short-CBP-KR12, at 11.72 and 10.43, respectively. The Long-CBP-KR-12’s isoelectric point, however, was just slightly below 10 at 9.84; this was still well above the 7.15–8.9 pH range reported for chronic wounds [59].

### 2.2. Free Peptide Cytotoxicity

**Figure 3:**
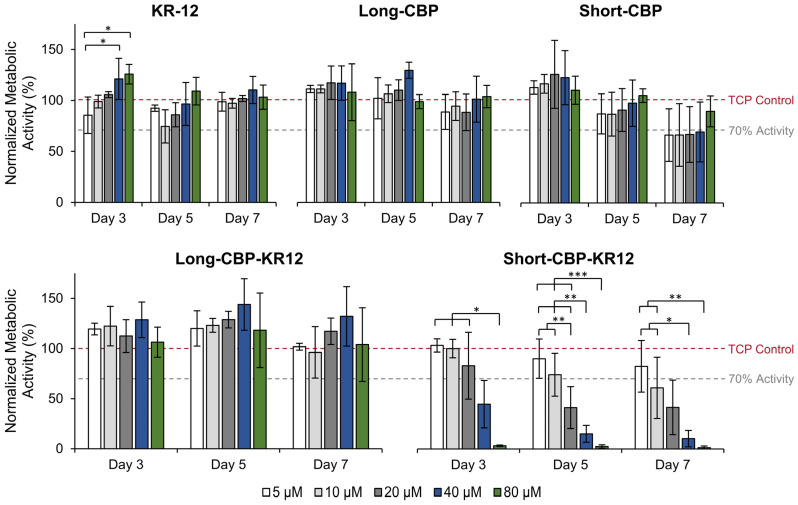
Solution peptide effect on NHDF viability assessed through metabolic activity. All data are normalized to the metabolic activity of untreated control cells indicated by the red dashed line; 70% activity is indicated by the grey dashed line. Data are presented as the mean and standard deviation from three independent experiments (*n* = 3 per experiment). Statistical differences determined by an ANOVA followed by the Tukey’s HSD test (* *p* < 0.05, ** *p* < 0.01, *** *p* < 0.005).

As the main proliferative cell type in the wound bed, NHDFs are the common engineering standard for in vitro cytotoxicity assessment according to ISO 10993-5 [64,65]. The metabolic effects of chimeric peptides and their individual component peptides in solution were assessed using NHDFs (Figure 3). Representative images were also compared to qualitatively assess cell morphology (Figure S1). Overall metabolic activity can be assumed to be proportional to cell count; thus, increases in metabolic activity compared to untreated controls can be perceived as increases in cell proliferation. The data are normalized to the untreated control, where 100% normalized metabolic activity indicates no change compared to the control (as indicated by the grey dashed line). At high KR-12 concentration on day 3, KR-12 resulted in increased metabolic activity at 40 µM (121% ± 20%) and 80 µM (125% ± 10%) compared to 5 µM (86% ± 18%). Though this trend in increasing proliferation in response to increasing KR-12 concentration was also observed at day 5, increased variability in the data resulted in no statistical significance. Knowing that KR-12 plays an active role in key wound healing pathways beyond antibacterial activity, the increased cell proliferation at higher concentrations agrees with findings from the literature [28]. Song et al. noted similar findings, where NHDFs exposed to higher levels of KR-12 exhibited increased metabolic activity and DNA content [29]. Further studies have indicated that no cytotoxicity towards human cells have been observed for concentrations greater than 63.5 µM [66]. Gunasekera et al. found that KR-12 showed weak cytotoxic activity towards a human lymphoma cell line resulting in a 13% loss in viability at an 80 µM concentration [67]. It should be noted that human lymphoma is not a cell line that KR-12 is known to act upon, explaining the cytotoxic effects rather than increased proliferative effects observed in this and previous work.

The Long-CBP exhibited no effects on metabolic activity at all concentrations across the 7 day period. As this CBP was originally designed by Khazanov et al. for use as a novel carrier for cellulose catalytic degradation, this is the first reported assessment of the cytotoxicity of the free peptide in vitro [38]. The Short-CBP exhibited no effect on fibroblast metabolic activity up until day 5. At day 7, decreased metabolic activity was observed across all Short-CBP concentrations with 5–40 µM resulting in a tight range of 67–70% normalized metabolic activity, while 80 µM resulted in 90% metabolic activity. It should be noted that similar trends were not observed for KR-12- or Long-CBP-treated cells, indicating that there may be some cytotoxicity induced by Short-CBP after prolonged exposure. No definitive conclusions could be drawn as increased cellular confluence and media depletion at later timepoints resulted in increased variability.

The Long-CBP-KR12, like its individual components, exhibited no effects on metabolic activity across all concentrations. The Short-CBP-KR12 peptide, however, exhibited increased cytotoxicity with concentrations at and above 20 µM. On day 3, 5 µM and 10 µM exhibited no significant effect on metabolic activity with 104% ± 6% and 101% ± 8%, respectively. Increasing peptide concentration to 20 µM resulted in a decrease in metabolic activity with a high variability of 44%. Increasing the peptide concentration above 20 µM resulted in statistically significant decreases in metabolic activity where 40 µM resulted in a metabolic activity of 45% ± 24% and 80 µM resulted in near complete cell death with a quantifiable metabolic activity of only 3% ± 1%. This trend was consistent for all time points evaluated and clearly observed in microscopy images (Figure S1). The increase in the cytotoxicity of Short-CBP-KR12, despite clear evidence of the biocompatibility of its individual CBP and AMP components, could be attributed to the low solubility of the peptide. Although Short-CBP-KR12 can initially solubilize in media, the peptide precipitated out of solution over the course of the initial 3 days, resulting in aggregation and increased cytotoxicity at the higher concentrations and prolonged exposure times. Solubility concerns are a known issue with AMPs in aqueous solution, and subsequent experiments were designed with the understanding that free Short-CBP-KR12 above 20 µM may result in adverse cellular responses [37]. This cytotoxic concentration is still higher than the reported cytotoxic threshold of soluble LL-37 at 13 µM [68]. According to ISO 10993-5, cytotoxicity is defined as any concentration that reduces the cellular metabolic activity by more than 30% compared to untreated control groups grown on tissue culture plastic (TCP) [37,65]. All peptide sequences except higher concentrations of Short-CBP-KR12 and later timepoints of Short-CBP fell well above the 70% relative metabolic activity cutoff.

### 2.3. Peptide Minimum Inhibitory Concentration (MIC)

**Figure 4:**
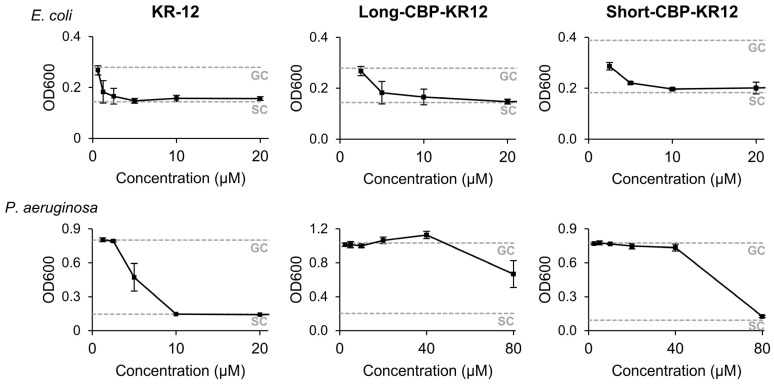
Optical density of *E. coli* and *P. aeruginosa* cultures in response to increasing concentrations of KR-12, Long-CBP-KR12 and Short-CBP-KR12, with untreated growth control values denoted as GC, and sterile control values denoted as SC. Data are presented as the mean and standard deviation from three independent experiments (*n* = 3 per experiment).

To assess if the antibacterial activity of KR-12 is maintained when contained within the chimeric peptide sequence, the minimum inhibitory concentration (MIC) of each KR-12-containing peptide was determined against Gram-positive *Staphylococcus aureus* (*S. aureus*), and Gram-negative *Escherichia coli* (*E. coli*) and *Pseudomonas aeruginosa* (*P. aeruginosa*) (Figure 4, Table S1). In this study, the MIC of KR-12 against *E. coli*, *P. aeruginosa*, and *S. aureus* were 2.5 µM, 10 µM, and >80 µM (not detectable), respectively. The calculated MIC against *E. coli* was comparable to the range of 2–2.5 µM previously reported [26,27,67]. The calculated MIC against *P. aeruginosa* was higher than the reported literature values of 4 µM [26]. *P. aeruginosa* is a bio-film-producing species that may hinder antibacterial activity through the AMPs’ normal mechanism of membrane interaction through electrostatic forces, resulting in increased MIC values [69]. Additionally, MIC values are variable as MIC values are highly dependent on initial inoculum concentrations and the MIC determination method. The mechanism of action of AMPs can be affected by membrane composition differences between bacteria strains and the reported MIC values can vary. For example, in the studies performed by Jacob et al., KR-12’s MIC against *S. aureus* KCTC-1621was reported as 4 µM; Luo et al. reported the MIC against *S. aureus* NCTC-6571 as 8.4 ± 6.3 µM; and Gunasekera et al. reported the MIC against *S. aureus* ATCC 29,213 as 4 µM [26,27,67]. Another beneficial aspect of KR-12 is its wide therapeutic index, where the antibacterial concentration is far lower than the concentrations that result in mammalian cytotoxicity. This was confirmed in this study where the highest calculatable MIC observed was 10 µM and mammalian cytotoxicity was not observed through 80 µM.

When assessing the antibacterial activity of both chimeric peptides, a decrease in overall activity was observed. Against *E. coli*, both the Long-CBP-KR12 and Short-CBP-KR12 inhibited bacterial growth at 10 µM, a four-fold increase in inhibitory concentration compared to KR-12. The inhibitory concentration for Long-CBP-KR12 against *P. aeruginosa* was determined to be greater than 80 µM, while the Short-CBP-KR12 peptide inhibited growth at 80 µM, an eight-fold increase compared to KR-12. This decrease in bioactivity was not unexpected. When linking collagen binding domains to LL-37, Lozeau et al. observed an almost 4-fold increase in inhibitory concentration against *E. coli* and an 11-fold increase against *P. aeruginosa* [23]. By including additional amino acids to the AMP, side interaction may hinder the effectiveness of the AMPs. Additionally, a reduction in the hydrophobic moment was identified in both the Long-CBP-KR12 and Short-CBP-KR12. Torres et al. determined that AMPs with hydrophobic moments above 0.6 and below 0.8 fall within a “hot spot” for favorable antibacterial activity [70]. The reduction in hydrophobic moment further explains the need for increased peptide concentration to inhibit bacterial growth. Overall, the antibacterial activity of the linked KR-12 in the designed chimeric peptides was maintained, and the Long-CBP-KR12 exhibited a wider therapeutic window than the Short-CBP-KR12.

### 2.4. Endotoxin Binding

**Figure 5:**
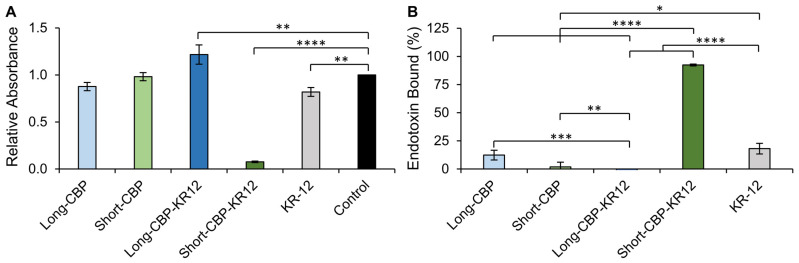
Endotoxin-binding activity of KR-12 and KR-12-containing chimeric peptides. (**A**) Endotoxin-binding capacity normalized to the untreated control group. Values below 1.0 indicate endotoxin binding and neutralization. Statistical differences determined by an ANOVA followed by a Dunnett HSD test comparing the means to the control group (** *p* < 0.005, **** *p* < 0.0001). (**B**) Normalized endotoxin binding converted to percent endotoxin bound. Statistical differences determined by an ANOVA followed by a Tukey’s HSD test (* *p* < 0.05, *** *p* < 0.001, **** *p* < 0.0001). Data are presented as the mean and standard deviation from three independent experiments (*n* = 3 per experiment).

LL-37 and KR-12 are able to interact with and mediate the inflammatory pathways stimulated by LPS via a neutralization mechanism [26,71]. LPS, embedded in the membranes of Gram-negative bacteria, is released upon bacterial cell death. Released LPS is known to stimulate toll-like receptor 4 (TLR-4), resulting in the activation of the downstream pro-inflammatory signaling cascade [26,68]. To investigate if the antiendotoxin activity in the chimeric peptides was retained, the LPS-binding capability of the peptides was evaluated. The binding capability was determined by assessing the peptide’s ability to inhibit the LPS-induced activation of the limulus amebocyte lysate (LAL) enzyme [26]. Peptide solutions were incubated with 3 EU/mL LPS and LAL enzyme activation was normalized to control wells treated with water that would not neutralize LPS (Figure 5A). In this setup, relative absorbance values close to 1 indicate no LPS binding, while values below 1 indicate binding. The Long-CBP and Short-CBP, with no known LPS-binding capacity, exhibited no statistical changes in LPS detection with relative absorption values of 0.88 ± 0.04 and 0.98 ± 0.04, respectively. Interestingly, the Long-CBP-KR12 exhibited an increase in detectable LPS resulting in an absorption value of 1.22 ± 0.1. Control wells with each peptide sequence were set up without LPS stimulation to assess if any LPS content was found in the synthetically synthesized peptides and could explain this increase in LPS detection. For all the peptides tested, no endogenous LPS was detected, indicating that the increased LAL enzyme activation was only present when Long-CBP-KR12 interacted with the spiked LPS and the LAL enzyme directly, though the exact mechanism is unknown and warrants further structural investigation. Both the Short-CBP-KR12 and KR-12 peptides exhibited a statistically significant decrease in LPS detection with absorption values of 0.08 ± 0.01 and 0.82 ± 0.05, respectively.

The relative absorbance values were converted to the bound endotoxin percentage (Figure 5B). KR-12 was shown to bind 18% ± 5% of LPS in solution. Though previously published LPS binding by KR-12 has not been reported above 10 µM, assuming that an increase in peptide concentration would increase binding, this value aligns with the previously reported 10% binding capacity at 10 µM [26]. Interestingly, though not statistically significant compared to the untreated control, the Long-CBP peptide was able to non-specifically bind to LPS (12% ± 4%). This is interesting as the CBP is neutrally charged and peptide–LPS interactions are believed to be driven by electrostatic interactions between positively charged peptides and negatively charged LPS. This non-specific interaction can be attributed to the hydrophobic interactions between the hydrophobic residues in the Long-CBP sequence and the lipid A domain of LPS, effectively producing peptide-LPS clusters that may prevent full LAL enzyme activation and detection [72,73]. This proposed “blocking” effect is different from the AMP interaction with LPS, which is described as inducing a structural change in the LPS, preventing LAL enzyme function [74,75]. Additionally, Rosenfeld et al. proposed that the ratio between hydrophobicity and charge is a strong determinant of LPS interactions [76]. Though the Short-CBP has a high hydrophobicity (Table 2), this same level of hydrophobic interaction is not observed, most likely owing to its shorter peptide [43]. When linked to KR-12, the Short-CBP-KR12 peptide reported high LPS-binding capacity (92% ± 1%). As the KR-12-containing peptide with the highest calculated hydrophobicity, this high LPS-binding capacity is likely due to a combination of AMP-LPS electrostatic and CBP-LPS hydrophobic interactions resulting in a chimeric peptide sequence that surpassed the LPS-binding capabilities of KR-12 alone.

### 2.5. Cellulose-Binding Capacity of the CBPs and Chimeric Peptides

The cellulose-binding capacity of Short-CBP, Long-CBP, and both chimeric peptides was assessed for KR-12 immobilization and the effect of the individual peptide regions on binding capacity. KR-12, with no specific cellulose-binding capacity, was used as a control. BC pellicles were prepared as previously described to produce opaque (0% arabitol) and transparent (85% arabitol) samples [36]. The binding assessment of opaque and transparent BC was performed based on the previously reported difference in cellulose fiber microstructure [36], which may impart differences in CBP binding. A previous use of the Short-CBP indicated increased binding selectivity to materials with fiber width in the µm range rather than the nm range [37]. Though not statistically significant, a marginal increase in binding capacity was observed for opaque BC as compared to transparent BC for all peptides tested.

**Figure 6:**
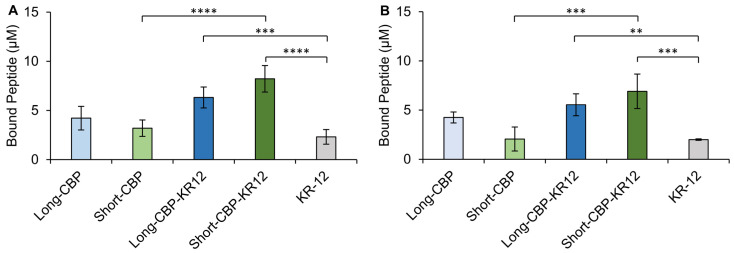
Bacterial cellulose-binding capacity of CBP-containing peptides. Peptide binding to (**A**) transparent and (**B**) opaque BC. Data are presented as the mean and standard deviation from three independent experiments (*n* = 3 per experiment). Statistical differences determined by an ANOVA followed by a Tukey’s HSD test (** *p* < 0.005, *** *p* < 0.001, **** *p* < 0.0001); to simplify presentation, only statistical differences between sample CBP peptides (e.g., Long-CBP vs. Long-CBP-KR12) and KR-12-containing peptides are annotated.

Binding was assessed through fluorescence emission of fluorescently tagged peptides in the supernatant and reported as peptide concentration bound to BC pellicles (Figure 6). Non-specific adsorption of KR-12 was observed on both the opaque and transparent BC at 2.3 µM and 2.0 µM, respectively. The surface of BC is believed to be slightly negative, allowing for electrostatic interaction between the positively charged AMP and the cellulose material. However, this interaction is not stable as fluorescence imaging of the KR-12-modified BC showed a decreasing signal over a 7-day period (Figures S2 and S3). Long-CBP and Short-CBP were observed to bind to both opaque (both at 4.2 µM) and transparent BC (3.2 µM and 2.1 µM, respectively). Increased binding on both opaque and transparent BC was observed for Long-CBP-KR12 (6.3 µM and 5.5 µM, respectively) and Short-CBP-KR-12 (8.2 µM and 6.9 µM, respectively) compared to their CBP only counterparts, although this difference was only statistically significant for the Short-CBP-KR12. Guo et al. determined that, as a short peptide with no tertiary structure, the Short-CBP has a reduced binding affinity to cellulose, but with the addition of additional amino acid groups binding affinity increases [43]. This similarity may explain why the Long-CBP with a more defined cyclized tertiary structure trends towards higher binding capacity than the Short-CBP. The Short-CBP-KR12 exhibited a trend towards increased binding capacity compared to the Long-CBP-KR12. When designing the Long-CBP, Khazanov et al. identified five main amino acid residues (Y5, Q34, Y32, N29, and Y31) participating in hydrogen bonding and hydrophobic interactions with the cellulose [38]. The Short-CBP, however, contains two amino acid residues (W3 and Y6) believed to be responsible for cellulose interactions [43]. It is possible that the smaller size and reduced cellulose interacting residues of the CBP in the Short-CBP-KR12 peptide allow for increased peptide per surface area coverage compared to the Long-CBP and resultant Long-CBP-KR12. Notably, all peptides containing a CBP sequence exhibited a consistent fluorescent signal over a 7-day period on both opaque and transparent BC. This indicated stable immobilization of the peptide using either the Long-CBP or Short-CBP. This prolonged binding was consistent with findings by Weishaupt et al. where the Short-CBP effectively immobilized active antimicrobial ligands for up to 21 days [37]. It should be noted that initial fluorescence intensities should not be compared as a measure of binding in the fluorescence images (Figures S2 and S3). The FAM-tagged peptides showed varying fluorescence intensities at the same peptide concentrations, likely due to hydrophobic interactions between the fluorescent tag and the more hydrophobic peptides [77]. Control images were taken of opaque and transparent BC, and no autofluorescence was observed.

### 2.6. Effect of Peptide-Functionalized BC on In Vitro Cytotoxicity

**Figure 7:**
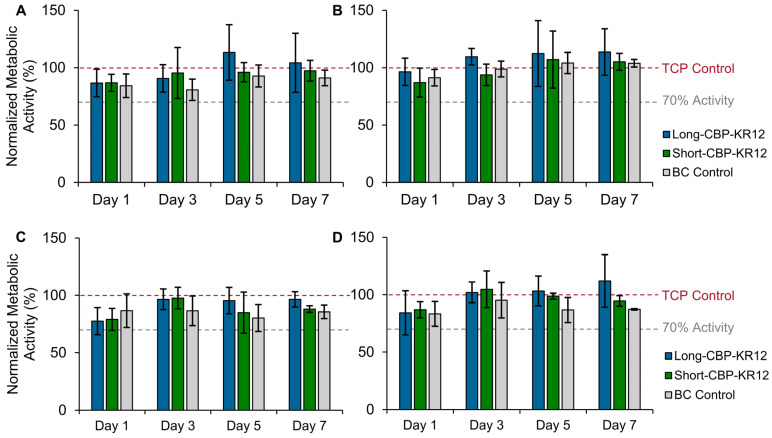
Functionalized BC effect on NHDF and HaCaT viability. Normalized metabolic activity of (**A**,**B**) NHDF and (**C**,**D**) HaCaT after exposure to unfunctionalized BC (BC control) and BC functionalized with Long-CBP-KR12 and Short-CBP-KR12 normalized to untreated cells. Both the (**A**,**C**) opaque and (**B**,**D**) transparent BC were evaluated. All data are normalized to the metabolic activity of untreated control wells indicated by the red dashed line, and 70% activity is indicated by the grey dashed line. Data are presented as the mean and standard deviation from three independent experiments (*n* = 3 per experiment). No statistical differences determined by ANOVA.

Cell proliferation and re-epithelialization of wounds is of great importance in wound care. Previously, BC’s biocompatibility and lack of effect on metabolic activity was shown for both transparent and opaque materials [36]. To assess the cytotoxicity of Long-CBP-KR12 and Short-CBP-KR12 functionalized opaque and transparent BC, metabolic activity assays were performed. Metabolic activity was assessed in both NHDF and HaCaT cells as the predominant proliferative cells present in the wound bed [29,45]. Unfunctionalized opaque or transparent BC was used as controls. No cytotoxicity was observed for any of the experimental and control groups, time points, and cell types evaluated (Figure 7). Considering the cytotoxic effects of soluble Short-CBP-KR12, the lack of cytotoxic response in mammalian cells exposed to Short-CBP-KR12-immobilized BC indicates limited peptide desorption from the BC. Consistent with previous findings, tethered KR-12 exhibited no cytotoxic effects across both cell lines and all time points. With the HaCaT cells, a slight increase in metabolic activity was observed in cells exposed to KR-12-functionalized BC. Though not as pronounced, this trend was consistent with prior findings by Song et al. and Liu et al. for HaCaT cell response to KR-12 covalently conjugated to silk and eggshell membrane materials [29,45]. The lower trend reported here can be attributed to the different methods of cell seeding. Both Song et al. and Liu at al. seeded on top of their functionalized material, allowing for more direct contact and interaction between KR-12 functionalized surfaces and cells. KR-12, like LL-37, is believed to activate the MAPK/ERK signaling pathway via transactivation of EGRF. This increases cell migration, proliferation, and differentiation and explains the increased metabolic activity [29,78,79]. Overall, these results indicate that BC functionalized with Long-CBP-KR12 and Short-CBP-KR12 not only had no negative effects on metabolic activity but may promote cell proliferation.

### 2.7. Surface Functionalized BC Antibacterial Activity

**Figure 8:**
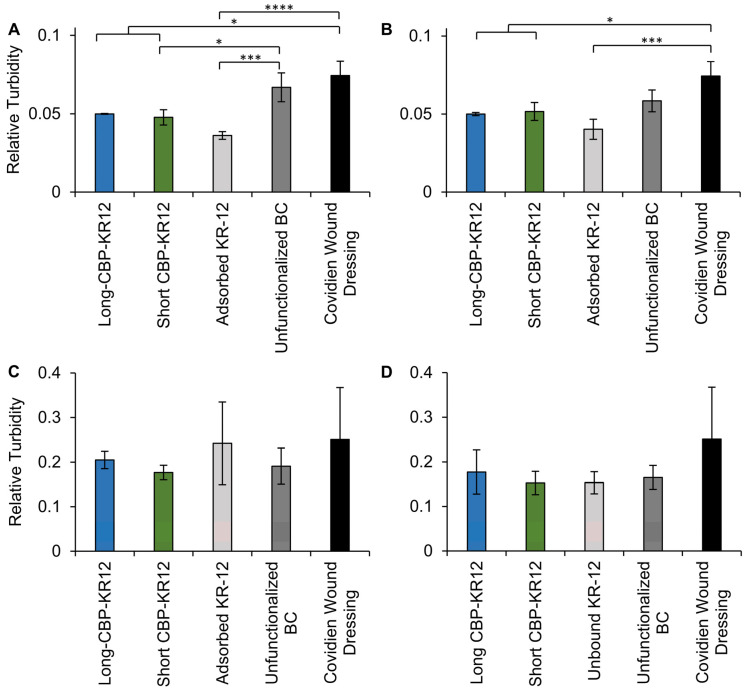
Relative turbidity of *E. coli* and *P. aeruginosa* incubated with functionalized BC. Relative turbidity of (**A**,**B**) *E. coli* cultures and (**C**,**D**) *P. aeruginosa* after functionalized BC exposure for 12 h. Both the (**A**,**C**) opaque and (**B**,**D**) transparent BC were evaluated. Data are presented as the mean and standard deviation from three independent experiments (*n* = 3 per experiment). Statistical differences determined by an ANOVA followed by a Tukey’s HSD test (* *p* < 0.05, *** *p* < 0.0005, **** *p*< 0.0001).

To evaluate the antibacterial activity of functionalized BC, the growth of *E. coli* and *P. aeruginosa* after 1 h exposure to Long-CBP-KR12 and Short-CBP-KR12 functionalized opaque and transparent BC were assessed. Functionalized BC pellicles were rinsed and allowed to incubate in fresh medium for 12 h before assessing bacteria growth via absorbance readings at 600 nm. Unfunctionalized BC and a commercially available hydrogel-based wound dressing with no known antibacterial activity were included as negative controls. BC loaded with KR-12 was included as a positive control. Though not statistically significant, a decreasing trend in *E. coli* growth was observed when comparing opaque functionalized and opaque unfunctionalized BC (Figure 8). However, this trend with *E. coli* was not observed for transparent BC. The Short-CBP-KR12 functionalized opaque BC resulted in a 29% reduction in *E. coli* growth compared to the unfunctionalized BC control. Compared to the hydrogel-based commercial wound dressing, opaque BC functionalized with Long-CBP-KR12 and Short-CBP-KR12 reduced *E. coli* growth by 33% and 36%, respectively. Similar trends were observed for functionalized transparent BC, where Long-CBP-KR12 functionalized transparent BC reduced *E. coli* growth by 33% and Short-CBP-KR12 functionalized transparent BC reduced *E. coli* growth by 31%. For both the opaque and transparent BC, adsorbed KR-12 reduced *E. coli* growth by at least 46% compared to the commercially available wound dressing, and upwards of 31% compared to unfunctionalized BC. With the MIC of KR-12 in solution being lower than either of the chimeric peptides, it is expected that the release of untethered KR-12 would result in bactericidal activity. However, KR-12 in solution is unstable in protease-rich environments, resulting in almost full degradation in 3 minutes and reduced longevity of the antibacterial activity [67]. Lozeau et al. reported similar finding for non-covalent peptide immobilization of LL-37, where free LL-37 reduced *E. coli* growth by ~65% while immobilized LL-37 reduced bacterial growth by either ~30% or ~60%, depending on the respective immobilization strategy [23]. No statistical difference in bacterial growth was observed across all functionalized and unfunctionalized materials tested against *P. aeruginosa*. This lack of antibacterial activity aligns with the higher minimum inhibitory concentration of the chimeric peptides against *P. aeruginosa* combined with the activity hindrance effects of immobilization [30].

Though the MIC of the free peptide provided a baseline prediction for the antibacterial activity of BC functionalized with each chimeric peptide, it is difficult to compare the effectiveness of free versus immobilized AMPs. This is due to a variety of factors that cannot be directly compared, such as surface density versus concentration in solution, and conformational freedom versus steric hindrance between free and immobilized AMPs [23,29]. Additional factors such as lack of precise quantification of immobilized AMPs further hinder direct comparisons [80]. In general, direct comparisons of free and immobilized AMPs are rarely described. Of the cases reported, immobilized AMPs resulted in increased MIC values between 8- and 64-fold higher than soluble AMPs [80,81,82]. Additionally, accurate assessments of reported immobilization effects on AMP activity are confounded specifically by the immobilization method and resulting steric hindrance [83]. Steric hindrance of the immobilized AMP poses a great impact on peptide bioactivity. For example, LL-37 directly conjugated to titanium fully restricted any bactericidal activity, but when immobilization occurred through a PEG linker, Gabriel et al. demonstrated that antibacterial activity was retained [84]. Further, Bagheri et al. demonstrated that immobilized AMP activity directly correlates to linker length and flexibility [85]. Similar to the work by Weishaupt et al., we utilized a flexible glycine-rich linker to reduce steric hindrance of the immobilized AMP [37,47]. Additional confounding factors present in literature comparison are the underreported immobilization of the C or N-terminal of the AMP. Previous findings for immobilized LL-37 reported immobilization through C-terminal modifications [16,23,24]. Similarly, Mishra et al. reported increased retention of the antibacterial activity of C-terminal immobilized LL-37 fragments which had a similar structure to KR-12 [49]. More recently, KR-12 immobilization through N-terminal covalent surface conjugation has been explored and heavily advocated for. Both Song et al. and Blasi-Romero et al. argue that due to the high density of hydrophobic residues found at KR-12’s C-terminal, covalent immobilization should occur at the N-terminal for retained antibacterial activity [29,30]. However, Bagheri et al. argues that AMP N- or C-terminal immobilization orientation is less relevant with the use of a flexible linker [85,86]. In the work reported here, KR-12 was immobilized via linker and CBP modification of the C-terminal. Overall, we observed a decreasing trend in bacterial growth of *E. coli* through KR-12 immobilization to opaque BC via CBP-driven cellulose surface interactions. This work outlines the first reported use of CBPs to tether KR-12 to BC materials. Although clinically relevant antibacterial activity was not achieved in this work, the effective immobilization and demonstrated activity retention of KR-12 indicates that CBP tethering is an overall promising approach to surface-functionalized antibacterial activity. To improve the overall antibacterial activity of surface-functionalized BC, alternative KR-12 analogs, backbone-cyclized KR-12 dimers, and chemically modified KR-12 could be explored [26,87,88].

## 3. Materials and Methods

### 3.1. Materials

*Komagataeibacter hansenii* NQ5 (*K. hansenii* ATCC 53582), normal human dermal fibroblasts (NHDFs; American Type Culture Collection: CRL-2565), *Staphylococcus aureus* (ATCC 43866, strain designation: LRA 44.01.83), *Escherichia coli* (ATCC 33694, strain designation: HB101) and *Pseudomonas aeruginosa* (ATCC 29260, strain designation: PA-103) were purchased from the American Type Culture Collection (ATCC), Manassas, VA, USA, while human endothelial keratinocyte cells (HaCaT, catalog number: T0020001) were purchased from AddexBio, San Diego, CA, USA. Bacto™ peptone and Difco™ yeast extract was purchased from Becton, Dickenson and Company, Franklin Lakes, NJ, USA, while agar was sourced from Sunrise Science Products, San Diego, CA, USA. D-arabitol (>99% purity), was purchased from Alfa Aesar Co., Inc., Ward Hill, MA, USA. D-glucose (BioReagent, ≥99.5% purity), citric acid (99% purity), sodium phosphate dibasic (BioReagent, ≥99.5% purity), lipopolysaccharides (LPS) from *Escherichia coli*, 2,2,2-Trifluoroethanol (TFE), resazurin sodium salt (BioReagent), Mueller–Hinton broth (MHB), and sodium hydroxide (ACS reagent, ≥97.0% purity), were sourced from Sigma-Aldrich, Burlington, MA, USA. Iscove’s modified Dulbecco’s medium was purchased from Lonza Biologics, Walkersville, MD, USA, HyClone Dulbecco’s Modified Eagle Medium (DMEM) with high glucose from Cytiva Marlborough, MA, USA, while fetal bovine serum (FBS), 0.25% trypsin-EDTA, penicillin streptomycin, and 200 mM L-glutamine were sourced from Life Technologies, Waltham, MA, USA. Animal-free recombinant human epidermal growth factor (EGF) and fibroblast growth factor (FGF) were purchased from PeproTech, Waltham, MA, USA. Tris-buffered saline (TBS) was sourced from VWR Life Sciences, Bridgeport, NJ, USA. All well plates were sourced from Greiner Bio-One, Monroe, NC, USA, while Falcon™ round-bottom test tubes, and endotoxin free dimethyl sulfoxide (DMSO) were sourced from Corning, Inc., Corning, NY, USA. Pierce^TM^ Chromogenic Endotoxin Quant Kit was purchased from Thermo Scientific. Waltham, MA, USA, and glacial acetic acid was sourced from J.T. Baker, Radnor Township, PA, USA.

### 3.2. Peptide Design

The following synthetic AMP and CBP peptides sequences were purchased from Biomatik Corporation (Canada) at >95% purity with an amidated C-terminus: KR-12 (KRIVQRIKDFLR), Short-CBP (WHWTYYW), and Long-CBP (**C**QVLNPWYSQTTPGWGQ**C**) with a disulfide bond between the cysteine residues [38,43]. Two different chimeric peptides were designed with the KR-12 peptide linked to either the Short-CBP or Long-CBP peptide via an inert flexible linker (GSGSGGS) (Table 3). A glycine and serine-rich linker was chosen to allow the two active domains to maintain rotational freedom, enhance the solubility of the hydrophobic peptides, and offer some resistance to proteolysis compared to other linker sequences [47,48]. The custom designed chimeric peptides were synthesized by CASLO ApS (Kongens Lyngby, Denmark). Amide group insertion at the C-terminal end of all peptide sequences occurred to provide proteolytic protection [89,90]. For spectrophotometric detection and quantification, all peptide sequences were purchased with a fluorescein (5,6-FAM) label attached via a mini-PEG linker. To ensure solubility, peptides were first reconstituted in dimethyl sulfoxide (DMSO), then further diluted in 1× TBS (25 mM tris, 140 mM NaCl, 3 mM KCl, pH 7.2), unless specified otherwise.

### 3.3. Peptide Modeling and Helix Wheel

Iterative Threading ASSEmbly Refinement (I-TASSER) server was used as an in silico method for predicting protein structure and function to qualitatively predict the secondary structure of each chimeric peptide sequence and its individual components [91,92,93]. Helix-wheel analysis was performed using the HELIQUEST platform (https://heliquest.ipmc.cnrs.fr/, accessed on 18 December 2023) to calculate the hydrophobic moment, amino acid location, and amphiphilicity of the peptides [61].

### 3.4. Circular Dichroism (CD) Spectroscopy

Circular dichroism spectroscopy (CD spec; JASCO J-1500, Jasco Inc., Oklahoma City, OK, USA) was used with modification from a previously published method [16]. Briefly, spectra analysis of 100 μM Long-CBP, Short-CBP, KR-12, Long-CBP-KR12, and Short-CBP-KR12 were obtained for wavelengths between 190 and 260 nm at 50 nm/min in a 10 mm path length cuvette at room temperature in 10 mM phosphate buffer, or buffer containing 0.1% LPS (representative environment) or 50% trifluoroethanol (TFE) solutions (positive control helix inducing solution). Thirty scans were recorded for each replicate (N = 3). The data were further analyzed for adopted secondary structure using the CDSSTR empirical model and reference set 7 in DichroWeb [62,63,94,95,96].

### 3.5. Mammalian Cell Culture Maintenance

Normal human dermal fibroblasts were maintained in Iscove’s Modified Dulbecco’s Medium supplemented with 10% FBS, 100 U/mL penicillin, 100 µg/mL streptomycin, 2 mM L-glutamine, 0.01 µg/mL EGF, and 0.005 µg/mL FGF). The HaCaT cell line, spontaneously transformed aneuploid immortal keratinocyte cell line, were maintained in Dulbecco’s Modified Eagle Media (DMEM) supplemented with 10% FBS, 100 U/mL penicillin, 100 µg/mL streptomycin, and 0.4 mM L-glutamine. All cells were maintained at 5% CO_2_ and 37 °C in a humidified environment. NHDFs were passaged using 0.25% trypsin-EDTA when 70–80% confluent, while HaCaT cells were passaged using both 0.25% trypsin-EDTA and cell scraping when 70–80% confluent.

### 3.6. Free Peptide Cytotoxicity

In a 96-well plate, 1000 NHDF cells were seeded per well and allowed to attach for 24 h. Medium was then replaced with fresh medium supplemented with each peptide in solution at 80, 40, 20, 10, 5, and 2.5 μM concentrations. On days 1, 3, 5, and 7 post-peptide treatment, a resazurin metabolic assay was performed to quantify cell metabolism. Fluorescent signals of the resazurin solution after incubation were read (excitation and emission at 544 nm and 590 nm) on a SpectraMax M2 plate reader (Molecular Devices, San Jose, CA, USA). Since the peptide was not expected to change the metabolic activity of the cells, fluorescent signal of resazurin directly correlated with the number of metabolically active cells in culture. For all time points, no-peptide treated cells were cultured on as a positive control. Resazurin solution was also added to wells with no cells as a negative control. For qualitative comparison, brightfield imaging of wells was performed on an upright microscope (Nikon Eclipse E600, Tokyo, Japan) with a digital camera (Spot Insight CMOS 5.1, Sterling Heights, MI, USA).

### 3.7. Peptide Minimum Inhibitory Concentration (MIC)

*S. aureus*, *E. coli*, and *P. aeruginosa* were cultured in Mueller–Hinton broth (MHB) and then maintained on Mueller–Hinton agar plates (MHA). Using a 0.5 McFarland Standard, cell turbidity was corrected to 1 × 10^8^ colony forming units per mL (CFU/mL) [97]. Cultures were diluted 100-fold in MHB to 1 × 10^6^ CFU/mL bacterial cultures. Diluted fresh cultures were used to determine the minimum inhibitory concentration (MIC) of Long-CBP, Short-CBP, KR-12, Long-CBP-KR12, and Short-CBP-KR12 in solution using a 96-well polypropylene plate based on standards set by the Clinical and Laboratory Standards Institute [23]. Briefly, serial diluted peptides starting at 80 μM concentrations were added to wells with 1 × 10^6^ CFU/mL fresh inoculum for each bacterial cell type, along with a no peptide control. Negative-control wells with no inoculum were also evaluated. Plates were incubated on a shaker and absorbance readings at 590 nm (OD590) were recorded using the Biotek Synergy H1 plate reader (BioTek Instruments Inc., Winooski, VT, USA) for 18 h after initial inoculation. The MIC was determined as the minimum peptide concentration that resulted in OD590 values not statistically different from sterile control values.

### 3.8. Endotoxin Binding

The endotoxin binding ability of the chimeric peptides was determined and compared to KR-12 using an end-point chromogenic limulus amebocyte lysate (LAL) quantification kit; method adapted from Wang et al. [98]. Peptide sequences were hydrated, diluted to 20 μM using endotoxin-free water and 25 μL were plated into round bottom polypropylene 96-well plates. An equal volume of 3 endotoxin units (EU)/mL LPS solution was mixed in each well and plates were incubated for 30 min at 37 °C to allow for peptide–LPS binding. Amebocyte lysate reagent was added, and the plates were further incubated for 10 min. The chromogenic substrate was added and incubated for 6 min and the reaction stopped using 25% acetic acid. The plates were spectrophotometrically analyzed at 405 nm using a SpectraMax M2 plate reader. Absorbance reduction as a function of peptide concentration is directly proportional to the neutralization of LPS in solution [26].

### 3.9. Cellulose Production

Bacterial-derived cellulose (BC) pellicles were produced as previously described [36]. Briefly, *Komagataeibacter hansenii* NQ5 static bacterium stock was established in Hestrin–Schramm (HS) medium (20 mg/mL glucose, 5 mg/mL peptone, 5 mg/mL yeast extract, 1.15 mg/mL citric acid, 2.7 mg/mL disodium phosphate). Fresh HS medium without a carbon source was then inoculated with 0.1% (*v*/*v*) static cultured bacteria and supplemented with either 1 mM glucose (opaque BC) or 0.15 mM glucose and 0.85 mM arabitol (transparent BC). To allow for pellicle formation, 200 μL of inoculated HS medium was cultured in 96-well tissue plates under static conditions at 30 °C for 7 d. To purify, the BC pellicles were transferred to 50 mL conical tubes, washed in 0.1 M NaOH at 60 °C for 4 h, rinsed with DI H_2_O until the fluid reached a neutral pH, and liquid autoclaved in 1× TBS.

### 3.10. Peptide Binding to BC

Due to the presence of residual proteins in the BC material and lack of tyrosine and tryptophan residues in the unaltered KR-12 peptide sequence, peptide quantification through BCA assay or 280 nm absorbance could not be performed. Quantification through fluorescent labeling or epitope tagging is well established in the field [23,37]. Working solutions of the fluorescently tagged peptides Long-CBP-FAM, Short-CBP-FAM, Long-CBP-KR12-FAM, Short-CBP-KR12-FAM, and KR-12-FAM peptide sequences were prepared in sterile 1× TBS to achieve a concentration of 20 μM. Individual opaque and transparent BC pellicles were placed in polypropylene 96-well plates with 200 μL of peptide solution. Plates were incubated on an orbital shaker at 37 °C for 48 h. Post incubation, BC pellicles were removed, washed with 1× TBS and used for further surface functionalization testing. Peptide binding was quantified through fluorescence analysis of the incubation supernatant (ex: 495 nm; em: 525 nm) using a SpectraMax M2 plate reader. Reduction in supernatant fluorescence compared to peptide control wells (with no BC added) correlates directly to peptide binding to BC. Peptide binding was compared between peptide sequences and between opaque and transparent BC pellicles.

### 3.11. Surface Functionalized BC Cytotoxicity

Opaque and transparent BC pellicles were produced and functionalized in 96-well plates as described in Section 3.11. Wells in 48-well plates were seeded with 3000 NHDF cells or 6000 HaCaT cells and the cells were allowed to incubate for 24 h. Functionalized and unfunctionalized BC were then placed in cell-seeded wells. On days 1, 3, 5, and 7 after adding BC pellicles, a resazurin metabolic assay was performed to quantify cell metabolism. Fluorescent signals of the resazurin solution after incubation were read (excitation and emission at 544 nm and 590 nm) on a SpectraMax M2 plate reader. For all time points, cells were also cultured on TCP with no BC added as a positive control, and resazurin solution was added to wells with no cells as a background control.

### 3.12. Surface Functionalized BC Bacterial Regrowth

Surface antibacterial activity was assessed against *E. coli* and *P. aeruginosa*. Functionalized and unfunctionalized transparent and opaque BC pellicles were synthesized and prepared as described in Section 3.11. BC pellicles were incubated in 200 µL of each type of bacteria at 1 × 10^8^ CFU/mL for 1 h in Mueller–Hinton broth (MHB), rinsed in sterile MHB to remove unattached bacteria, and incubated with 200 µL fresh MHB at 37 °C for 12 h. Bacterial regrowth was determined by comparing relative turbidity (absorbance at 600 nm) readings on media incubated with functionalized and unfunctionalized BC. This procedure was also repeated on a commercially available hydrogel-based wound dressing material with no known antibacterial activity.

### 3.13. Statistical Analysis

All statistical analysis was performed using GraphPad Prism 7. Statistical analysis of the adopted secondary structure, effects on metabolic activity, minimum inhibitory concentration, LPS binding, cellulose binding, and effects on turbidity were conducted using an analysis of variance (ANOVA) followed by either a Tukey or Dunnett’s post hoc test, as specified in the figure caption. All data are presented as mean ± standard deviation (SD) unless otherwise indicated.

## 4. Conclusions

In this study, we designed chimeric peptides to non-covalently modify the surface of two forms of BC, opaque and transparent, by linking bioactive CBPs with KR-12 and assessed the overall bioactivity of the chimeric peptides. As the secondary structure of AMPs is believed to be essential for bioactivity, conservation of the α-helix structure of KR-12 was initially predicted in silico and then confirmed experimentally through CD spectroscopy of both the Long-CBP-KR12 and Short-CBP-KR12. Chimeric peptides and their individual components were assessed for cytotoxicity, where only higher concentrations of Short-CBP-KR12 were studied and longer timepoints of Short-CBP-KR12 exposure exhibited negative effects on metabolic activity, which were attributed to solubility issues. Additionally, trends in increasing metabolic activity in response to increasing KR-12 and Long-CBP-KR12 concentrations were observed. All KR-12-containing peptides exhibited antibacterial activity in solution against *E. coli* and *P. aeruginosa*, though higher inhibition concentrations were required for both chimeric peptides, likely due to the fact that the chimeric peptides were predicted to exhibit changes in antibacterial activity-mediating properties such as hydrophobic moment and hydrophobicity. The Short-CBP-KR12 peptide exhibited enhanced LPS-binding capabilities compared to both the KR-12 control and Long-CBP-KR12. Both Long- and Short-CBP-binding mechanisms allowed effective binding and stable immobilization of Short-CBP-KR12 and Long-CBP-KR12 over a 7-day period. Reduced antibacterial activity of immobilized Short-CBP-KR12 and Long-CBP-KR12 was observed, which is consistent with previous findings. Additionally, all functionalized materials exhibited no adverse effects on the metabolic activity of both NHDF and HaCaT epithelial cells. The results from this study support the effectiveness of CBP-driven immobilization of AMPs and warrant further investigation into the wound-healing capabilities of non-covalently immobilized KR-12.

## Figures and Tables

**Table 1:** Secondary structural predication by I-TASSER server based on peptide sequence.

Peptide Label	Sequence/Predicted Secondary Structure
**Long-CBP**	GSGSGGSCQVLNPWYSQTTPGWGQC CCCCCCCCSSSCCHHCCCCCCCCCC
**Short-CBP**	GSGSGGSWHWTYYW CCCCCCCSSSSSSC
**KR-12**	KRIVQRIKDFLR CHHHHHHHHHHC
**Long-CBP-KR12**	KRIVQRIKDFLRGSGSGGSCQVLNPWYSQTTPGWGQC CHHHHHHHHHHCCCCCCCCCSSCCHHHHCCCCCCCCC
**Short-CBP-KR12**	KRIVQRIKDFLRGSGSGGSWHWTYYW CHHHHHHHHHHCCCCCCCCSSSSSSC

H: Helix; S: Strand; C: Coil.

**Table 2:** Physiochemical properties of CBPs, AMPs, and chimeric peptide sequences.

Peptide Label	Mw (Da) ^a^	pI ^a^	q ^b^	AI ^a^	Hx (%) ^c^	µH ^b^	H ^b^	II ^a^
**Long-CBP**	2557.75	5.51	0	27.2	4.3	0.286	0.480	24.72
**Short-CBP**	1630.70	6.74	0	0	3.3	0.128	0.639	23.63
**KR-12**	1571.93	11.72	+4	121.67	56.0	0.782	0.193	31.95
**Long-CBP-KR12**	4111.67	9.84	+4	57.84	69.0	0.387	0.387	25.04
**Short-CBP-KR12**	3184.61	10.43	+4	56.15	53.0	0.346	0.435	24.59

Abbreviations: Mw, molecular weight; pI, isoelectric point; q, charge; AI, aliphatic index; Hx, helicity; µH, hydrophobic moment; H, hydrophobicity; II, instability index. ^a^ calculated with EXpasy Protparam [60]. ^b^ calculated with the HeliQuest platform [61]. ^c^ calculated with DichroWeb from CD spectra [62,63].

**Table 3:** Peptide labels and sequence designation.

Peptide Label	Peptide Sequence
Short-CBP	WHWTYYW-NH_2_
Long-CBP	CQVLNPWYSQTTPGWGQC-NH_2_
KR-12	KRIVQRIKDFLR-NH_2_
Short-CBP-KR12	KRIVQRIKDFLR-GSGSGGS-WHWTYYW-NH_2_
Long-CBP-KR12	KRIVQRIKDFLR-GSGSGGS- CQVLNPWYSQTTPGWGQC-NH_2_
Short-CBP-FAM	WHWTYYW-(mini-PEG)-K(5-FAM)-NH_2_
Long-CBP-FAM	CQVLNPWYSQTTPGWGQC-(mini-PEG)-K(5-FAM)-NH_2_
KR-12-FAM	KRIVQRIKDFLR-(min-iPEG)-K(5-FAM)-NH_2_
Short-CBP-KR12-FAM	KRIVQRIKDFLR-GSGSGGS-WHWTYYW-NH_2_
Long-CBP-KR12-FAM	KRIVQRIKDFLR-GSGSGGS-CQVLNPWYSQTTPGWGQC-(mini-PEG)-K(5-FAM)-NH_2_

## Data Availability

The datasets generated and/or analyzed during the current study are included in this published article and its Supplementary Materials.

## Supplementary Materials

The following supporting information can be downloaded at: https://www.mdpi.com/article/10.3390/ijms25031462/s1.

## Abbreviations

AI	Aliphatic index
ANOVA	Analysis of variance
ATCC	American Type Culture Collection
AMPs	Antimicrobial peptides
BC	Bacterial cellulose
CBD	Cellulose-binding domain
CBP	Cellulose-binding peptide
CD	Circular dichroism
DMEM	Dulbecco’s Modified Eagle Medium
DMSO	Dimethyl sulfoxide
ECM	Extra cellular matrix
EU	Endotoxin units
FBS	Fetal bovine serum
GC	Growth control
H	Hydrophobicity
Hx	Helicity
HaCaT	Human epidermal keratinocytes
HS	Hestrin–Schramm
HSD	Honest significant difference
II	Instability index
I-TASSER	Iterative Threading ASSEmbly Refinement
LAL	Limulus amebocyte lysate
LPS	Lipopolysaccharide
MHA	Mueller–Hinton agar
MHB	Mueller–Hinton broth
Mw	Molecular weight
MIC	Minimum inhibitory concentration
MRSA	Methicillin-resistant *Staphylococcus aureus*
NHDFs	Normal human dermal fibroblasts
PEG	Poly(ethylene glycol)
PI	Isoelectric point
q	Charge
ROS	Reactive oxygen species
SD	Standard deviation
SC	Sterile control
TBS	Tris-buffered saline
TCP	Tissue culture plastic
TFE	Trifluoroethanol
µH	Hydrophobic moment
