# Supplementary material for: Functionalization of Bacterial Cellulose with the Antimicrobial Peptide KR-12 via Chimerical Cellulose-Binding Peptides
Source: Int J Mol Sci. 2024 Jan 25;25(3):1462. doi: 10.3390/ijms25031462 (PMC10855235; doi:10.3390/ijms25031462)
Supplement: Supplementary file 1 [file ijms-25-01462-s001.zip › ijms-2808298-supplementary.pdf]

SUPPLEMENTARY MATERIALS

Functionalization of Bacterial Cellulose with the Antimicrobial Peptide KR-12 via Chimerical Cellulose Binding Peptides

Elizabeth M. van Zyl[a], Jeannine M. Coburn[a,*]

[a] Department of Biomedical Engineering, Worcester Polytechnic Institute, Worcester, MA 01609

Elizabeth M. van Zyl
emvanzyl@wpi.edu
Department of Biomedical Engineering
Worcester Polytechnic Institute
100 Institute Road
Worcester, MA 01609
United States of America

Jeannine M. Coburn
*Corresponding author
jmcoburn@wpi.edu
Department of Biomedical Engineering,
Worcester Polytechnic Institute
Ph: 508-831-6839
100 Institute Road
Worcester, MA 01609
United States of America

Total Pages: 5

Figures: 3

Tables: 1

Figure S1. Representative images of NHDF on Day 3 after treatment with peptides.
Figure S2. Images of FAM-tagged peptides immobilized on opaque BC over 7 days.
Figure S3. Images of FAM-tagged peptides immobilized on transparent BC over 7 days.
Table S1. Minimum inhibitory concentration (MIC) of KR-12 containing peptides against the three bacteria species/strains evaluated.

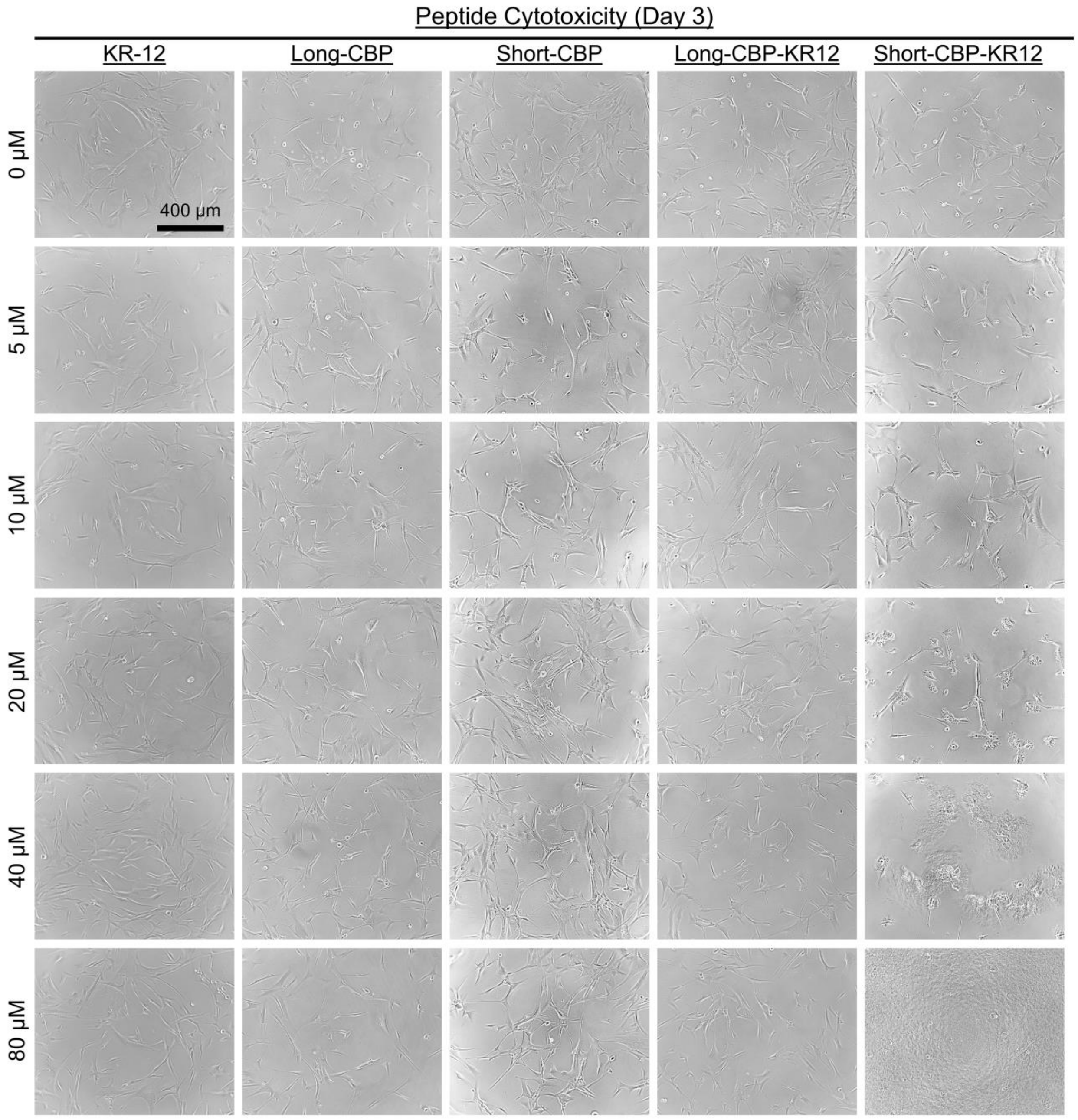

Figure S1. Representative images of NHDF on Day 3 after treatment with peptides.

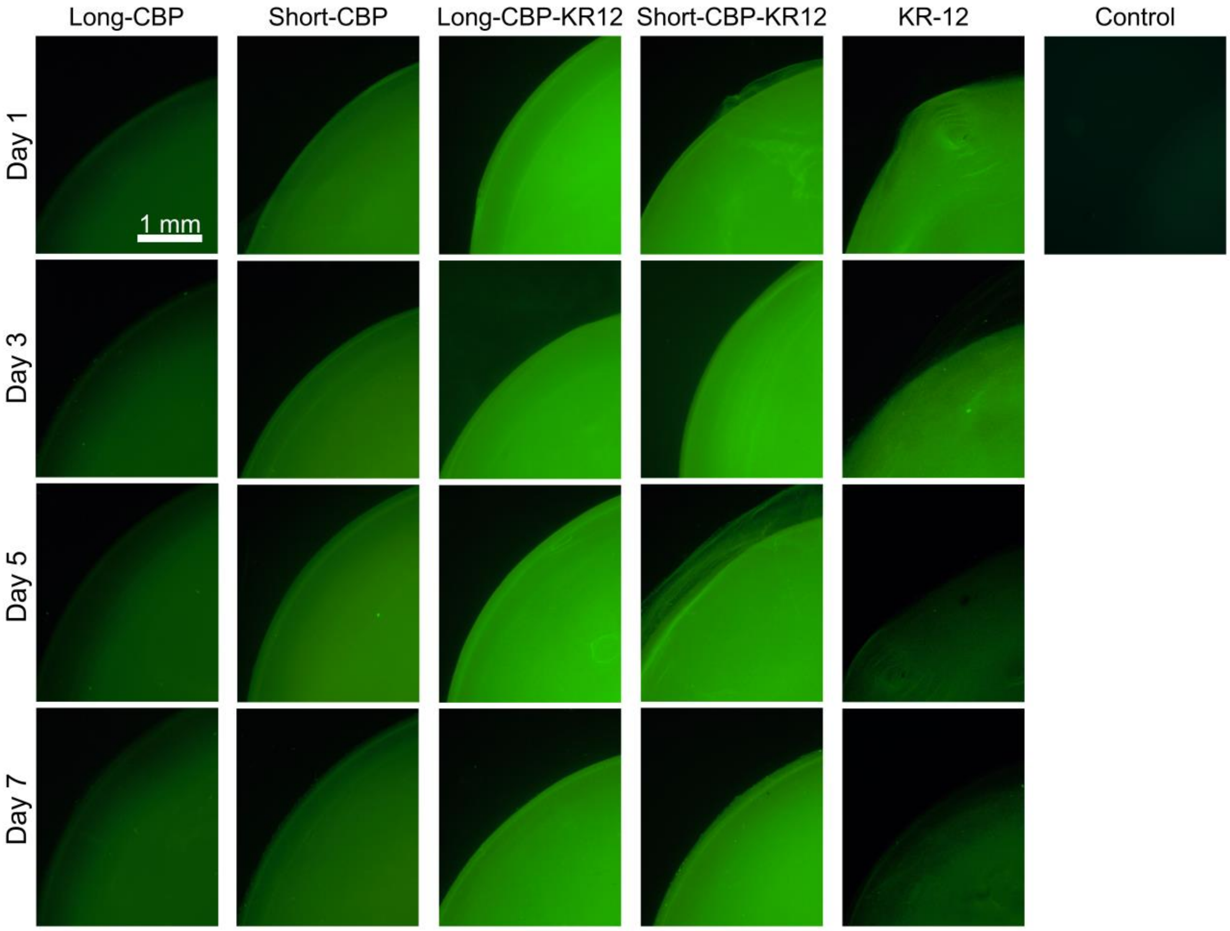

Figure S2. Images of FAM-tagged peptides immobilized on opaque BC over 7 days. Qualitative images of FAM-tagged peptide present on the surface of opaque 0% arabitol BC. Images were taken on days 1, 3, 5, 7 after initial rinsing. The gain and exposure time settings were kept constant for all images. The control image of unmodified BC shows no autofluorescence.

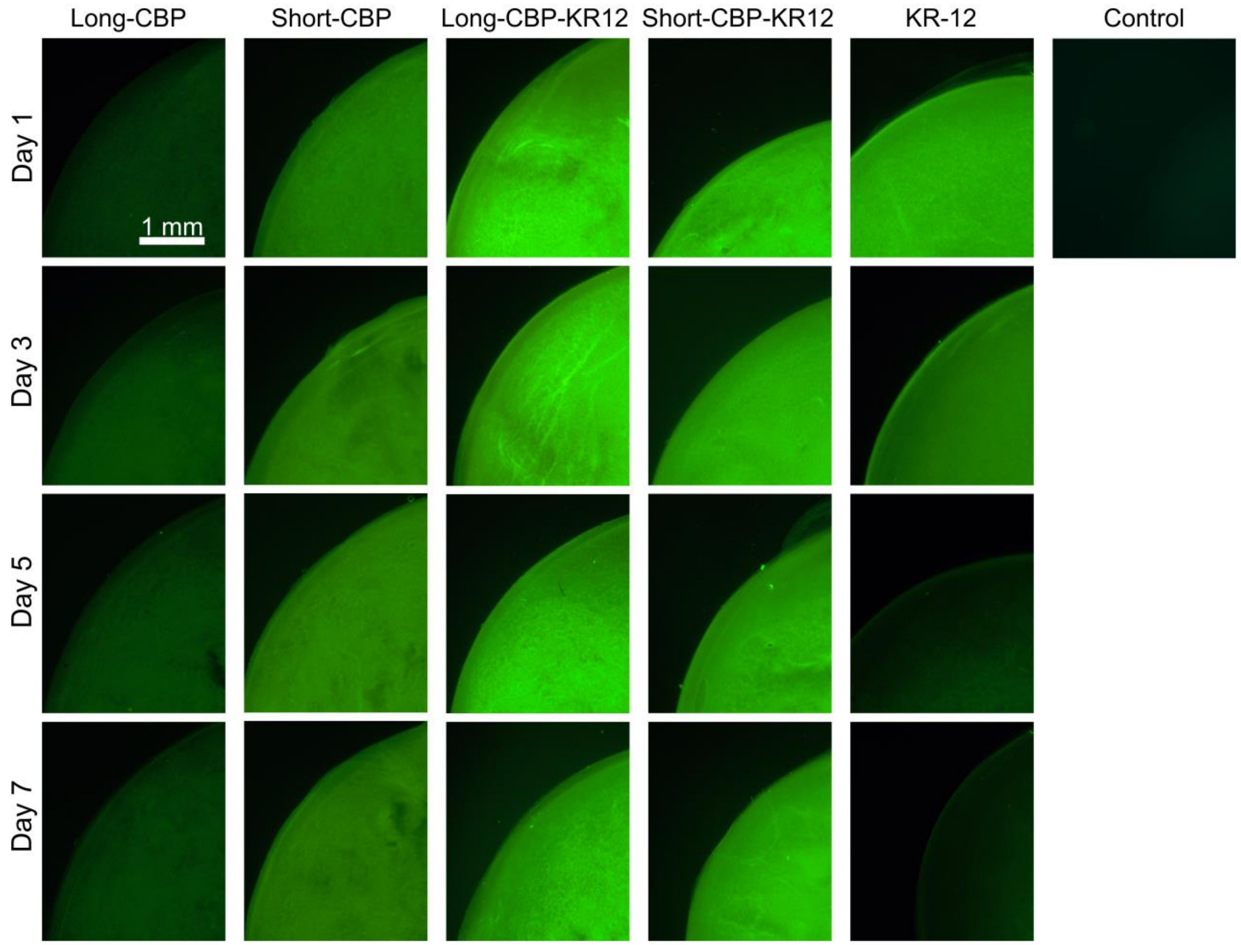

Figure S3. Images of FAM-tagged peptides immobilized on transparent BC over 7 days. Qualitative images of FAM-tagged peptide present on the surface of transparent BC. Images were taken on days 1, 3, 5, 7 after initial rinsing. The intensity, exposure time, and gain settings were the same for all images. The control image of unmodified BC shows no autofluorescence.

Table S1. Minimum inhibitory concentration (MIC) of KR-12 containing peptides against the three bacteria species/strains evaluated.

**Bacterial Species**	**Minimum Inhibitory Concentration (μM)[a]**		
	KR-12	Long-CBP-KR12	Short-CBP-KR12
Escherichia coli	2.5	10	10
Pseudomonas aeruginosa	10	>80	80
Staphylococcus aureus	>80	-	-

[a] Minimum inhibitory concentration (MIC) determined as the lowest concentration to not be statistical significance compared to the sterile control as determined by an ANOVA followed by a Dunnett HSD test.
